# Editorial: Adverse Pregnancy Outcomes—A Missed Opportunity to Prevent Chronic Disease?

**DOI:** 10.3389/ijph.2021.582810

**Published:** 2021-01-25

**Authors:** Peter M. Barrett

**Affiliations:** ^1^School of Public Health, Western Gateway Building, University College Cork, Cork, Ireland; ^2^ Irish Centre for Maternal and Child Health Research, University College Cork, Cork, Ireland; ^3^ Wellcome Trust/HRB Irish Clinical Academic Training (ICAT) Programme, University College Cork, Cork, Ireland

**Keywords:** pregnancy, kidney, cardiovascular disease, maternal health, prevention

**The IJPH series “Young Researcher Editorial” is a training project of the Swiss School of Public Health**.

In recent years, evidence has been mounting that adverse pregnancy outcomes are associated with increased risk of maternal chronic diseases in later life. Large-scale cohort studies and comprehensive meta-analyses have linked preeclampsia, gestational hypertension, preterm delivery, and pregnancy loss with higher risk of later cardiovascular disease (CVD) and chronic kidney disease (CKD) (1–4). Women who experience pregnancy complications are at greater risk of adverse cardiometabolic and renal outcomes than those who experience uncomplicated pregnancies. These associations are concerning from a population perspective. Every year, about 210 million women become pregnant, resulting in 140 million live births (5). The incidence of adverse pregnancy outcomes is rising along with maternal obesity, older age at conception, and higher prevalence of pre-pregnancy comorbidities (3, 6).

The links between adverse pregnancy outcomes and maternal CVD and CKD suggest that women’s pregnancy history may include information that could help prevent chronic disease. Recent guidelines from the American College of Cardiology state that pregnancy complications may be regarded as “risk-enhancing factors” for CVD, particularly among women whose 10-years cardiovascular risk is intermediate or low (7). For example, women who have had adverse pregnancy outcomes may benefit from more intensive lifestyle modifications for primary prevention of CVD than women whose pregnancies were uncomplicated. Similarly, women at intermediate risk of CVD may benefit from earlier commencement or higher doses of statin therapy if they have a history of preeclampsia (7). Obstetric information could be used by both healthcare providers and women affected by pregnancy complications to prevent onset or progression of CVD and CKD in later life. To make use of this information, however, we must meet three key challenges.

First, current research suggests that clinicians are often unaware of the associations between adverse pregnancy outcomes and chronic disease (8, 9). Few general practitioners and internal medicine physicians report asking parous women their obstetric history when assessing their risk of CVD beyond their reproductive years. Obstetricians are often more aware of these risks, but they do not typically follow up women after discharge from maternity services (8). Insufficient awareness might be caused by 1) lack of accessible evidence-based guidelines for monitoring women with adverse pregnancy outcomes over the long-term; 2) different and competing versions of published guidelines; and 3) obstetric and primary care services may not share electronic healthcare records (8, 9). Any of these problems may make healthcare professionals uncertain about whose clinical responsibility it is to inform affected women of their long-term risk (10).

Second, women who experience adverse pregnancy outcomes may be unaware of their individual risk of chronic disease (10, 11). Patients and clinicians may have different perceptions of the importance of personalized information. In Canada, some doctors assumed that women who had preeclampsia did not want to know their long-term CVD risk if these women did not inquire directly about this after pregnancy (10). But in Norway, women who had experienced pregnancy complications wanted to be given more individualized information about their future cardiometabolic risk by default (11). If neither healthcare providers nor patients initiate discussion about the links between adverse pregnancy outcomes and chronic disease, this prevention opportunity may be missed.

Third, the optimal timing, format and content of structured follow-up programmes for women with adverse pregnancy outcomes is unclear. Some have proposed systematic screening programmes (e.g., for postpartum hypertension, albuminuria, or other markers of cardiometabolic or renal disease) to prevent or diagnose CVD and CKD early on. But we do not yet know if the long-term clinical benefits of screening outweigh harms that could arise from over-investigation. We also do not know if adverse pregnancy outcomes add incremental value to existing risk prediction tools for CVD and CKD in parous women (1). Despite these limitations, healthcare providers should be encouraged to discuss the links between adverse pregnancy outcomes and CVD/CKD risk with their patients because this knowledge may motivate and empower women to change other modifiable risk factors for chronic disease. It may also help them make informed decisions about uptake of preventive interventions or treatment options (7).

Adverse pregnancy outcomes are a sex-specific suite of risk markers for chronic disease that offer a unique opportunity to reduce health inequities. CVD is the leading cause of mortality among women, but they are less likely than men to receive appropriate preventive care for CVD (12). CKD prevalence is higher in women than in men, affecting about one in eight women over their life-course (1, 13). From a global perspective, mothers in lower-income countries disproportionately suffer from pregnancy complications and the burden of chronic disease is growing in the Global South (5, 6). If pregnancy-related information can be used to prevent CVD and CKD, this could reduce some of these disparities.

From a clinical perspective, we can now shift our focus from asking if there are associations between pregnancy complications and chronic diseases to asking how we can harness this information to increase prevention. More healthcare providers will understand these risks if adverse pregnancy outcomes are consistently incorporated into clinical guidelines for preventing CVD and CKD, and if these guidelines are accessible in both community and hospital settings (9). At the regional level, obstetricians, general practitioners, and internal medicine physicians should collaborate to ensure that affected women are told of their heightened risk of chronic disease, and to maximize opportunities for providing preventive healthcare. Finally, at the health system level, chronic disease prevention strategies should be integrated with efforts to improve maternal health across the life-course. Taking a synergistic approach could lower the burden of chronic disease and ensure more equitable health outcomes for women over the long-term (5, 6).
